# Pressure-Sensitive Paint Measurements of Transient Shock Phenomena

**DOI:** 10.3390/s130404404

**Published:** 2013-04-02

**Authors:** Mark Kenneth Quinn, Konstantinos Kontis

**Affiliations:** Aero-Physics Laboratory, George Begg Building, University of Manchester, Sackville Street, Manchester, M13 9PL, UK; E-Mail: k.kontis@manchester.ac.uk

**Keywords:** pressure-sensitive paint, PSP, TLC, unsteady, transient, diffraction, shock waves, shock tube

## Abstract

Measurements of the global pressure field created by shock wave diffraction have been captured optically using a porous pressure-sensitive paint. The pressure field created by a diffracting shock wave shows large increases and decreases in pressure and can be reasonably accurately captured using CFD. The substrate, a thin-layer chromatography (TLC) plate, has been dipped in a luminophore solution. TLC plates are readily available and easy to prepare. Illumination comes from two high-intensity broadband Xenon arc light sources with short-pass filters. The sample is imaged at 100 kHz using a Vision Research Phantom V710 in conjunction with a pair of long and short pass filters, creating a band. The PSP results are compared with numerical simulations of the flow using the commercial CFD package Fluent as part of ANSYS 13 for two Mach numbers.

## 1. Introduction

A recent review on pressure-sensitive paint (PSP) technologies was conducted by Kontis [1] in which the scope and measurement range of PSP was highlighted. Several examples were presented of measurements of steady state high-speed flow using PSP. Unsteady high-speed pressure measurements using PSP represent a significant challenge.

Unsteady pressure measurements have been carried out using pressure-sensitive paint (PSP) by several researchers [2–9]. However, this is slightly misleading, as the term *unsteady flow* covers both repeating signals (such as those from a fluidic oscillator) and transients, such as a passing shock wave. Only two of the papers mentioned, Asai *et al.* [2] and Gongora-Orozco *et al.* [8], measured the pressure distribution globally of a transient flow. Both researchers used a shock tube to generate a transient pressure field, and attempted to measure the surface pressure globally. Asai *et al.* used a shock tube with a partially evacuated driven section and a pressurised driver section. This sub-atmospheric starting pressure gives PSP a higher sensitivity, regardless of the substrate used [10]. Gongora-Orozco *et al.* used a shock tube with an atmospheric driven pressure to capture a shock moving over grooves and successfully showed the modified shape of the shock front. Shock tubes are an excellent method of delivering predictable step changes in pressure, allowing us to look at the response times of the PSP. The aim of this work is to build upon the foundation laid by Asai *et al.* and Gongora-Orozco *et al.* and globally measure the pressure of a complex transient shock interaction at a baseline of atmospheric pressure. Shock wave diffraction around a large corner gives large positive and negative pressure changes and has been studied using a variety of other flow diagnostic techniques [11–13], meaning that this flow is relatively well understood. This work aims to investigate shock wave diffraction at two Mach numbers and compare the results with numerical data.

## 2. Background

### 2.1. Shock Tube

**Figure 1.**
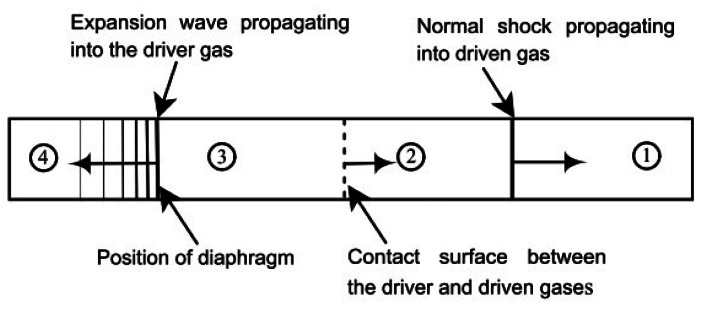
Shock tube flow after the diaphragm is ruptured.

Shock tubes are an ideal method to test the response time of an experimental technique. A region of high pressure (region 4) is separated from a region of low pressure (region 1) by a diaphragm. The diaphragm in the University of Manchester square shock tube is made up of acetate, which is burst by mechanical means. After the rupture of the diaphragm, compression waves begin to travel from region 4 into region 1. These compression waves eventually coalesce into a discontinuous shock wave. The pressure at each location in the wave structure shown in Figure 1 can be analysed using the one-dimensional theory presented by Anderson [14].

### 2.2. Shock Diffraction

**Figure 2.**
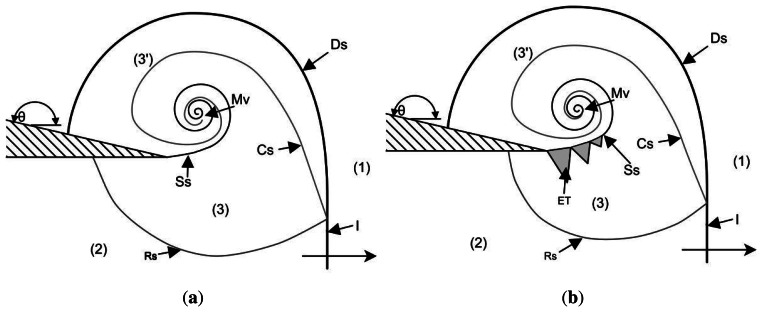
Basic flow structure behind a shock wave diffracting around a sharp corner. (**a**) 1 < *M_ie_* ≤ 1.35 incident shock wave; (**b**) 1.35 < *M_ie_* ≤ ≈ 1.8 incident shock wave.

The problem of shock wave diffraction around sharp corners has been investigated by several researchers since initial considerations by Howard and Matthews [15]. Many experimental (all previous experimental work used density-based diagnostics) and numerical simulations have been performed, showing different levels of flow features. The basic structure of a strong shock wave diffracting around a corner was given by Skews [16]. As a shock wave reaches a sharp corner with an angle greater than 75°, the flow is independent of geometry and only a function of incident shock Mach number. The diffracting shock wave loses strength along its length as it rounds the corner. The flow behind the incident shock (termed the “complex region” by Skews [16]) consists of a shear layer created by the inability of the flow to navigate the sharp corner. This shear layer rolls up into a spiral vortex (see Figure 2).

Sun and Takayama [17] showed that the lambda shock found underneath the shear layer appears after a critical incident shock Mach number of M*_i_* = 1.346. The pressure rise generated by the incident shock and the pressure decrease found at the centre of the shed spiral vortex make this flow an excellent test case for high-speed PSP measurements. Two different Mach numbers will be tested and compared with numerical simulations, namely experimental Mach numbers of M*_i_* = 1.28 and M*_i_* = 1.55. The expected flow schematic for these two Mach numbers is given in Figure 2.

In Figure 2, **I** is the incident shock, **Ds** is the diffracted shock, **Rs** is the reflected expansion wave, **Cs** is the contact surface, **Ss** is the shear layer, **Mv** is the main vortex and **ET** is a train of expansion waves and shocks. The bracket numbers represent regions of flow with different properties as they have been affected by different waves. Region (1) is the quiescent region ahead of the incident shock; (2) is a region of uniform flow behind the incident shock wave; (3) is the region affected by the reflected expansion wave and region (3′) has been affected by diffracted (curved) portion of the incident shock and as such has a lower pressure.

### 2.3. PSP

The basic theory of intensity-based pressure-sensitive paint has already been covered in depth by many authors; as such, a full recollection is not warranted here. For information on the theoretical background of pressure-sensitive paint, the reader is directed to the excellent book by Liu and Sullivan [18].

PSP is based on the mechanism of oxygen quenching, which involves the non-radiative deactivation of an excited photo-active molecule (luminophore). A luminophore is excited to an electronic state higher than its ground state by absorbing light of a specific wavelength. This excited luminophore can return to its ground state by either a radiative or non-radiative process. Radiative processes, which involve the emission of light, include fluorescence and phosphorescence and are usually grouped together under the term luminescence. The wavelength difference between the absorbed and emitted light is known as the Stokes shift [19]. It is a preferable characteristic that PSP luminophores have a large Stokes shift to allow the excitation and emission signals to be separated easily. Non-radiative processes include internal conversion to a different electronic state and then the release of heat, or external conversion via contact with an external molecule, in this case oxygen. Oxygen is an extremely good quenching molecule, as it has an unusual electronic ground state that is easily excited [20].

**Figure 3.**
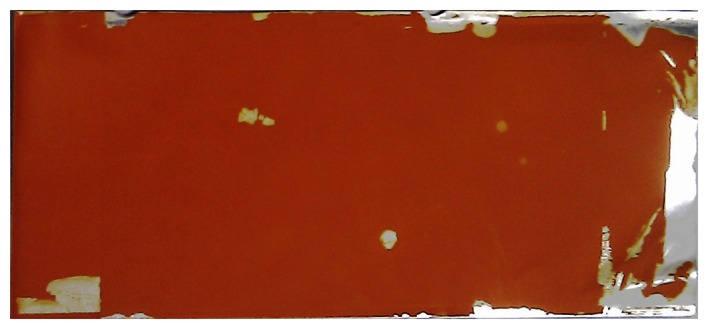
Mechanical damage to several initial TLC plates after 10 runs of the shock tube.

The method of application of pressure-sensitive paint, specifically the substrate to which it is applied, is critical as the response times can vary by 6 orders of magnitude. For example, the polymer formulation used by Carroll *et al.* [21] has a 90% response time of 480–2,500 ms whereas the anodised aluminium substrate used by Sakaue and Sullivan responds in under 0.4 ms [4]. An investigation into the performance of different substrates was performed by Quinn *et al.* [10]. Their results showed that, of the porous substrates, thin-layer chromatography (TLC) plates gave the highest signal output and sensitivity. However, TLC plates are extremely fragile and are only suited to very simple geometries. In such a violent environment as a shock tube, the TLC plates begin to fracture after approximately 10 individual runs (see Figure 3), limiting their repeated use. Uniformity of the PSP surface is critical in PSP measurements, as any inhomogeneities can produce strong differences between wind-on and -off images. Also, Figure 3 shows that areas of the silica gel substrate have fractured off, leaving no PSP present, meaning no signal can be measured in this region.

The relationship between intensity ratio and pressure ratio is given by an allometric formulation of the Stern–Volmer equation [10]:

(1) $$\frac{I_{\text{ref}}}{I}=A_{\text{Freundlich}}(T)+B_{\text{Freundlich}}(T){\left(\frac{P}{P_{\text{ref}}}\right)}^{\gamma }$$

where *I* is intensity, *P* is pressure, *γ*, *A* and *B* are experimentally determined constants and values with the subscript *_ref_* denoting a reference condition. The temperature dependency of the coefficients *A* and *B* is not expected to be a significant problem during this experiments as the testing time is so short (< 1 ms) that the heat transfer to the surface is almost negligible.

## 3. Numerical Simulation

Numerical simulations were performed using the commercial CFD code Fluent as part of ANSYS 13. A grid dependency study and solver discretisation study were both performed in order to determine the effect of numerics on the results. The simulation is initialised at atmospheric pressure everywhere, with pressure outlet conditions at edges of the test sections. A sponge layer is included in the mesh to avoid wave reflections from the pressure outlet boundaries. The driver section is then patched at the required pressure to generate the desired speed of shock wave based on inviscid theory.

The simulation used an inviscid, density-based solver, which was 2nd-order discretised in both space and time. The ASUM+ flux vector splitting scheme was chosen as it is known to perform well when resolving shocks [22]. An initial uniform regular structured grid consisting of quadrilateral 0.4 × 0.4 mm elements was then adapted 4 times per time-step based on the pressure gradient, giving a minimum Δ*x* = 25 *μ*m. This led to a maximum grid size of ≈ 300 K cells. The CFL number was kept at 0.2, as it was found that numbers larger than this gave non-physical oscillations in the region of strong shocks. This very strict CFL criterion suggests that Fluent is a highly non-diffusive code and, if controlled properly, is well suited to problems of this nature. The simulations took approximately 2 weeks to complete on an Intel core I7 desktop PC with 8 GB of RAM running Windows 7 (64 bit). The baseline mesh was generated using Gambit and post-processing was performed using Tecplot360.

## 4. Experimental Setup

**Figure 4.**
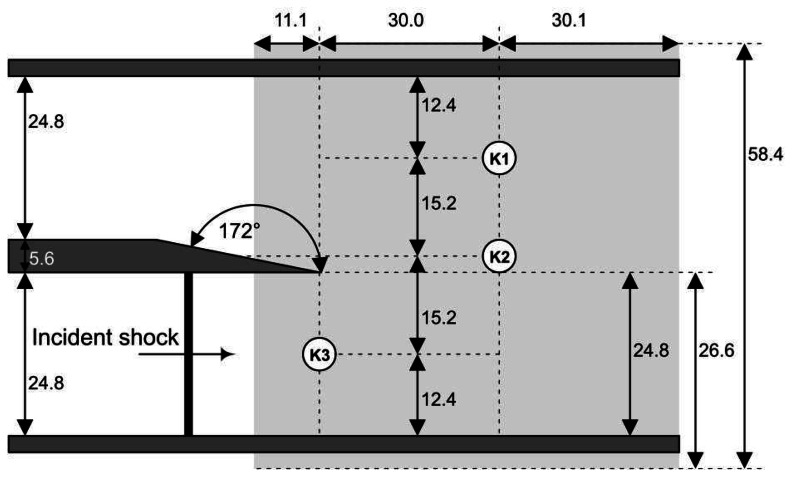
Test section geometry (all dimensions in mm).

The experiments shown here were carried out in the University of Manchester Aero-Physics Laboratory using the recently refurbished square shock tube. This mechanical rupture-style shock tube has a 24.8 × 24.8 mm cross-section square tube with a 700 mm driver section and a 1700 mm driven section. The height of the test section is 55.2 mm. A schematic of the test section, which is made of 10 mm UV transmitting Perspex, is shown in Figure 4. The geometry tested has a knife-edge tip and a wedge angle of *θ* = 8°. The driven and test sections are left open to atmosphere with only the driver section being pressurised.

Given an incident shock wave Mach number, *M_ie_*, we can calculate the pressure ratio across the shock, *P*_2_/*P*_1_, using inviscid theory. The speed of the shock waves is measured very accurately (the uncertainty in Mach number is ±0.02) by creating an x-t diagram using shadowgraph results captured at 250 kfps. Based on the experimental Mach number of the incident shock wave, the pressure ratio across a M*_ie_* = 1.28 shock wave is 1.75, while *P*_2_/*P*_1_ for M*_ie_* = 1.55 is 2.64. Given that the atmospheric pressure in the lab is almost exactly 1 bar, the expected pressures behind the incident shock waves are 1.75 and 2.64 bar respectively.

### 4.1. Transducer Measurements

The driver section pressure was measured using a pressure scanner (Druck PDCR820-0800) connected to a signal amplifier (Fylde 254 GA MINI AMP). High-speed pressure measurements were taken using three flush-mounted (Wheatstone-bridge based) pressure transducers (Kulite XT190-M) in the locations shown in Figure 4. The Kulite transducers were connected to an in-house-built signal conditioner to amplify their millivolt outputs. The natural frequency of these transducers is 380 kHz, which limited the safe acquisition speed to 100 kHz at most. The amplifier, which is made up of a series of operational amplifier circuits, has a low-noise linear gain of 250 up to a frequency of 900 kHz. To keep costs down, the amplifier had a fixed gain; however, the offset voltage could be adjusted using a potentiometer. The amplified signals are recorded by a data acquisition system (NI-USB6251 16-bit, 1.25 MS/s M Series Multifunction DAQ). The DAQ was connected to a HP Windows 7 Laptop running Lab View 2011. The LabView code, written by the author, monitored the driver section pressure until the desired limit was reached. At this point the software sent the acquisition tasks to the hardware using low-level VI functions rather than the Express-Vis built into LabView, as they are restricted in their timing to the Windows clock speed. The DAQ takes an analogue input as a trigger, meaning that a fourth XT190-M could be used at a distance (1 m) from the end of the shock tube to trigger acquisition. The trigger signal was connected to a Photonics Four Pulse Sequencer (SEQ400), allowing the user to set a hardware delay.

### 4.2. Sample Preparation

In order to deposit the luminophore onto a surface, first it must be dissolved in solution. Sakaue [23] showed the difference between ten solvents with widely varying polarity. The two most promising solvents for dissolving $\text{Ru}{\left(\text{dpp}\right)}_{3}^{2+}$ are dichloromethane and acetone as these deposited the most luminophore on the surface, therefore producing the highest signal level. Dichloromethane gave the highest pressure sensitivity over a large range; however, acetone gave the highest signal output.

It is known that the higher the concentration of luminophore in solution, the more will be deposited on the surface until the surface is saturated. However, Sakaue and Ishii [24] showed that increasing the amount of luminophore deposited on the surface does not monotonically increase the signal output or the sensitivity; rather, it decreases past a critical value. They found that the optimum luminophore concentration is of the order of 0.1 mM. Sakaue and Ishii [25] also showed that the length of time for which the substrate is immersed in the luminophore solution has a strong effect on the signal output and the temperature sensitivity of the final sample. They found the optimum time to be approximately 17 min. A rigorous test of the effect of dipping duration was not conducted in this study, although initial samples were dipped for 5, 10, 15 and 20 min. Longer dipping times resulted in significant solvent evaporation during the dipping process, meaning the concentration of the solution was no longer constant. Samples dipped for less than 15 min gave an insufficient signal output, whereas the 15 and 20 min samples were almost indistinguishable. It was decided to keep the dipping time consistent with the study by Sakaue and Ishii. The authors acknowledge that the ideality of this time does not necessarily extend from one substrate to another, although the rough initial tests showed it was a suitable value.

Using the two basic formulae related to molar concentrations allows for the calculation of the required mass of luminophore in a given volume of solvent for a specific concentration. 64.85 mg of $\text{Ru}{\left(\text{dpp}\right)}_{3}^{2+}$ (purchased from GFS chemicals) was dissolved in 100 mL of dichloromethane (purchased from Sigma Aldrich) at room temperature, giving a 0.5 mM solution. (Initial attempts with lower concentrations gave an unsatisfactory signal output.) The TLC plate (MERCK HPTLC Silica-Gel 60) has been used previously by Gongora-Orozco *et al.* [26], who showed the difficulties that can occur using the dipping method of application. If great care is taken, uniform application can be achieved using this method, as can be seen in Figure 3 (excluding the mechanical damage due to testing). The samples were placed in the solution for 17 minutes and then placed in an oven at 340 K to evaporate the solvent, leaving a uniform coating of luminophore molecules on the porous substrate.

Preparation of PSP samples in this way should be performed using safety apparatus, such as high-grade filter masks to prevent inhalation of the dichloromethane fumes. Thick safety gloves should be worn when handling the samples, as the solution can begin to dissolve parts of skin tissue.

#### 4.2.1. PSP Setup

**Figure 5.**
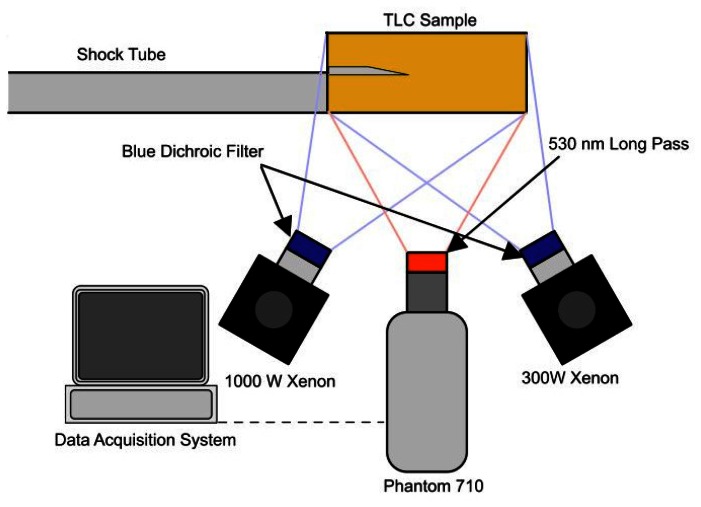
PSP setup.

**Figure 6.**
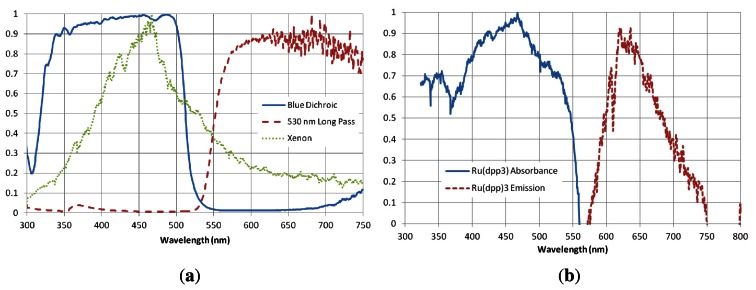
Spectral measurements of filter combination and PSP sample. (**a**) Filters and light source spectra; (**b**) $\text{Ru}{\left(\text{dpp}\right)}_{3}^{2+}$ absorption and emission spectra.

The high-speed PSP system used in this research is shown in Figure 5. Two blue dichroic filters were used in conjunction with heat absorbent glass to filter the broadband excitation sources. The excitation light sources are a 1, 000 W and 300 W xenon arc lamps. The 1,000 W lamp required a water filter to remove excess heat from the beam to avoid damage to filters. The emission signal was filtered using a 530 nm long-pass (*i.e.*, a filter passing only wavelengths longer than 530 nm) filter attached to a Vision Research Phantom 710 high-speed video camera. Gongora-Orozco *et al.* showed the importance of choosing the correct filter combination for PSP tests and how the signal level can be severely affected by this choice [27]. The spectra of the light source and the relevant filter transmissions were measured using the Princeton Instruments ICCD spectrometer and are given in Figure 6, along with the absorption and emission spectra of $\text{Ru}{\left(\text{dpp}\right)}_{3}^{2+}$. The Phantom 710 is well suited for tests of this type, due to its large pixel size and high quantum efficiency (quantum efficiency can be considered a measure of the sensitivity of the imaging device and is dependent on wavelength) over a wide range of wavelengths. The capture rate was 100 kHz with an exposure time of 9.6 *μ*s. At such a high frame rate, the image resolution is limited to 224 × 184 pixels, giving a pixel density of approximately 3.2 pixels per mm. The field-of-view of the PSP experiment is shown as the grey shaded area in Figure 4. All of the PSP and CFD results have the origin at the tip of the splitter geometry. Triggering of the capture was performed using a further XT190-M. The decision not to use a delay generator was taken so that the images taken before the arrival of the shock wave can be averaged and used as the reference, wind-off image.

There are two conventional methods of calibrating pressure-sensitive paints: *in situ* and *a priori*. *In situ* calibration involves using pressure taps and transducers on the actual test model in order to verify the response of the paint. This increases the cost and complexity of the model—the very thing PSP is designed to reduce. Initial *a priori* calibrations of the TLC samples (with such powerful light sources) showed significant photodegradation over the calibration process, meaning that the calibration constants were not reliable. Also, as the humidity changes from day to day, the PSP test response will change; however, the calibration will not. Therefore, it was decided to use *in situ* calibration. Although *in situ* will not prevent photodegratation, it will mitigate its effects as the image acquisition takes place over 1 ms, meaning that even a 50% per hour degradation rate will cause an insignificant change over the measurement time.

The pressure measurements taken using the Kulite transducers are used to calibrate the intensity ratio at the tap location. This type of unsteady calibration means that instead of the usual 10 or so pressure taps used for *in situ* calibration, three taps over 50 images can be used, giving 150 discrete calibration points.

**Figure 7.**
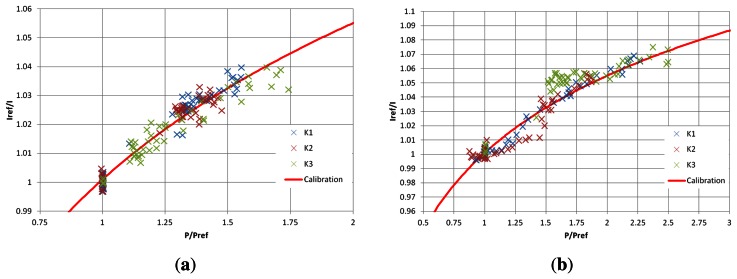
*In situ* calibration of PSP samples. (**a**) $\frac{I_{\text{ref}}}{I}$ *vs.* $\frac{P}{P_{\text{ref}}}$, *M_ie_* = 1.28 Sharp geometry; (**b**) $\frac{I_{\text{ref}}}{I}$ *vs.* $\frac{P}{P_{\text{ref}}}$, *M_ie_* = 1.55 Sharp geometry.

The intensity ratio from the PSP images is plotted against the pressure ratio measured by the Kulite transducers in Figure 7. The trend between the two follows the expected curve given by Equation 1 and is consistent with previous results seen by the authors [10]. Using the signal processing software OriginPro, a curve fit for the data is estimated to give the calibration constants for each test (Table 1). The small values seen for the exponent *γ* are due to the pressure ratio range being significantly larger than the intensity ratio change. The spread around the curve fit seen in Figure 7 is almost completely due to the measurement of the optical signal from the PSP. This can be seen by examining the *x* = 1 location on these graphs where the spread is purely vertical. Measuring optical emission signals with such low exposures as 10 *μ*s leads to a relatively low signal-to-noise ratio, hence the R^2^ values of approximately 0.93. This is highlighted in Table 2 where the shot noise is clearly dominant.

## 5. Results and Discussion

### 5.1. PSP Response

**Figure 8.**
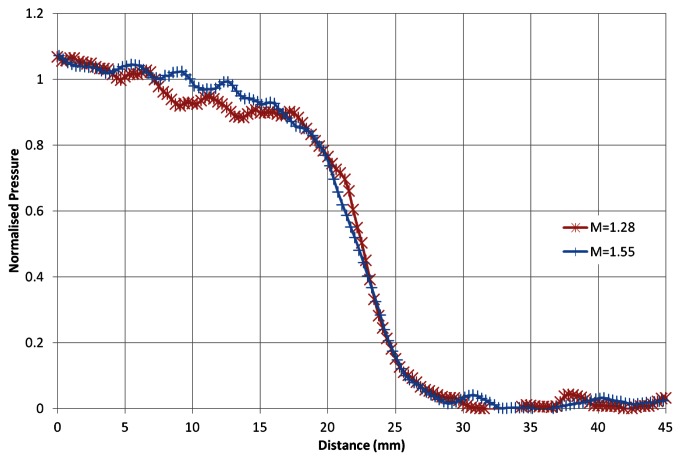
Normalised response of the PSP to two different shock waves.

Figure 8 shows the response of the PSP layer to two shock waves of different strength. This figure was generated by vertically averaging 20 pixels. The pressure rise is normalised to the value given by inviscid theory based on the experimental Mach number (normalised pressure values of 0 indicate that the shock has not yet arrived at that location). The x-axis corresponds to the distance from the left of the image to the end of the region of interest. The response is almost identical for both shock waves. The rise to a normalised value of 0.9 takes place over approximately 12 mm. The spread of the pressure rise corresponds to the distance travelled by the shock in approximately 20 *μ*s.

It is not possible to measure the response time any more accurately than this, as the image acquisition rate is 100 kHz. Initially there is a very shallow response of the PSP. This is due to the finite exposure time of the image blurring the shock front. Based on the shock speed we can calculate that the estimated blur over the exposure time is approximately 4.4 mm and 5.3 mm for a Mach 1.28 and 1.55 shock, respectively. After this initial slow rise, the paint responds well, showing a sharp increase in pressure that slowly begins to tail off just after 90%. Spatial resolution of the PSP should also be considered. Liu and Sullivan [18] stated that the spatial resolution of the PSP is approximately 5 times the sensor layer thickness due to sideways diffusion through the layer. MERCK quote that the thickness of the silica-gel is 200 *μ*m, meaning that, based on this estimate, the spatial resolution is 1 mm (3.2 pixels). Based on Figure 8, the spatial resolution of the PSP (and acquisition system) seems adequate to capture the shock front, as the response shows a smooth curve, highlighting that this is not the limiting factor.

### 5.2. M_i_ = 1.28 Results

#### 5.2.1. Transducer Results

**Figure 9.**
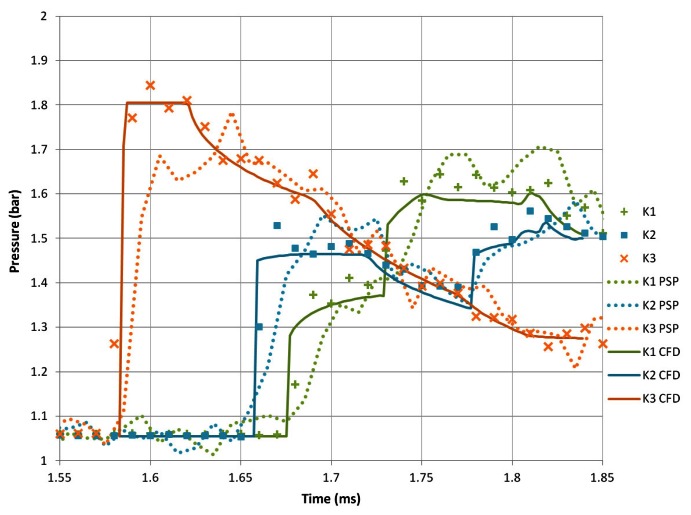
Pressure transducer measurements of M*_i_* = 1.28 shock diffraction process around sharp geometry.

Figure 9 shows the pressure history along the side wall of the shock tube test section as measured by the Kulite transducers, the PSP and the CFD simulations. **K3** is the first transducer to be influenced by the flow and gives a pressure rise to a maximum of 1.84 bar. Pressure, then, decays as the reflected expansion wave propagates upstream. **K2** shows a very sharp pressure rise 50 *μ*s later but only to 1.53 bar. This lower pressure peak is due to the diffracted shock wave being weaker than the incident one from which it spawns. **K2** also shows a rise in pressure, a slow decay and then another rise. This pattern is due to the initial shock and then the expansion region underneath the shear layer. The second pressure rise is almost certainly the passage of the returning diffracted shock, which has reflected off the top wall of the test section. **K1** is the last transducer to be affected and shows a two-stage pressure rise to a maximum of 1.63 bar. This is due to the diffracted shock wave reflecting off the top surface of the test section and therefore compressing the flow over this transducer twice.

The virtual transducers extracted from the numerical simulations agree well with the results obtained from the experimental transducers. The rises and falls are well predicted in terms of both time and magnitude. The simulations are able to resolve the incident shock well, with no discernible overshoot in pressure. Obviously the numerical transducers will have a smoother profile as they were taken at each time step in the simulation, whereas the Kulite transducers cannot be used reliably past 100 kHz. The PSP measurements take slightly longer to reach their maximum values. The initial pressure rise behind the incident shock is under predicted by PSP corresponding to the outliers in the calibration plots seen previously.

The transducers do show some level of spatial noise. This noise is likely due to the well-known ringing effect of Wheatstone bridge transducers that are subjected to a step function.

#### 5.2.2. PSP Results

**Figure 10.**
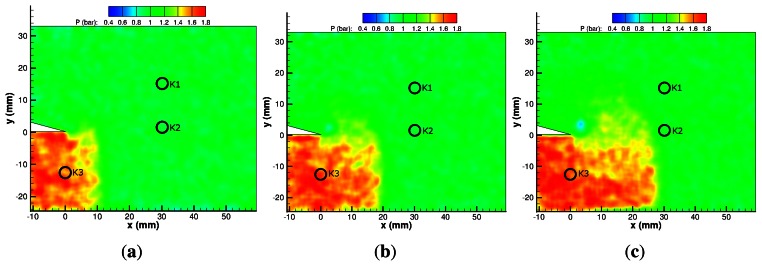

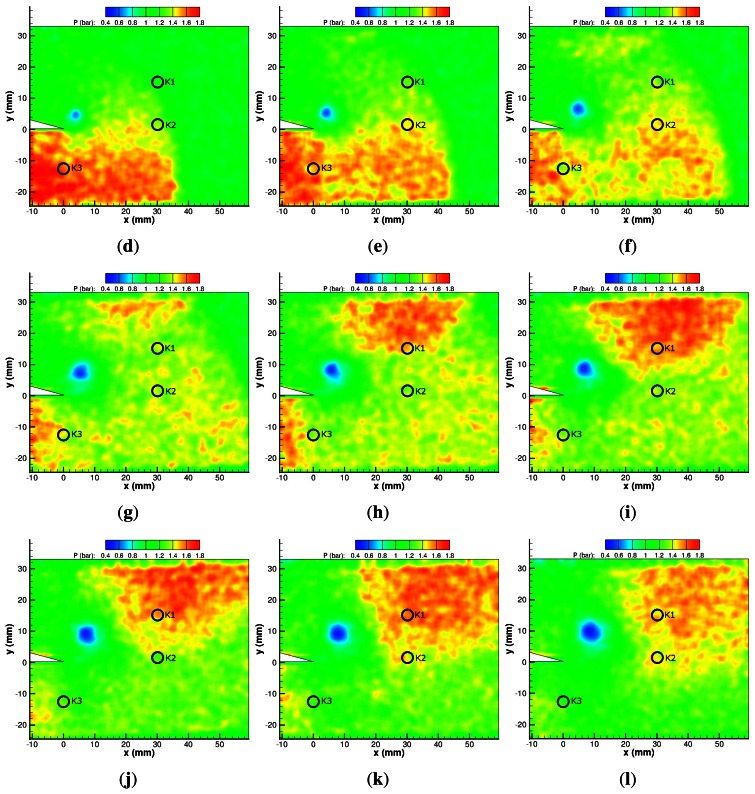
PSP map of M*_i_* = 1.28 shock diffraction process. (**a**) 20 *μ*s; (**b**) 40 *μ*s; (**c**) 60 *μ*s; (**d**) 80 *μ*s; (**e**) 100 *μ*s; (**f**) 120 *μ*s; (**g**) 140 *μ*s; (**h**) 160 *μ*s; (**i**) 180 *μ*s; (**j**) 200 *μ*s; (**k**) 220 *μ*s; (**l**) 240 *μ*s.

Figure 10(a) shows the incident shock wave and the pressure rise behind it. There is a significant degree of spatial noise despite taking an ensemble average of three individual tests and using a 3 × 3 linear filter to process the results. The pressure rise of 1.68 bar correlates well with the expected value of 1.75 bar given by inviscid theory based on M*_ie_*. Figure 10(b) begins to show the vortex shed from the apex. Subsequent, Figure 10(c) and onwards show the pressure drop in the centre of the vortex to its lowest value of 0.46 bar. The vortex remains approximately the same size from Figure 10(f) onwards. In the same images, the reflected expansion wave can be seen to be propagating upstream and downwards from the apex, as the pressure in this region is reduced compared with the region of uniform high pressure at the inlet to the test section. The pressure above the splitter does not change significantly over the first few images, indicating that the diffracted shock wave in this region is extremely weak.

Figure 10(e) shows the diffracted shock just as it is returning from the top wall. There is a small pressure rise behind it, which is more clearly seen in Figure 10(f). The pressure rise is not uniform along the length of the returning shock wave as it is barely noticeable on the left hand side of the vortex and significantly stronger on the right hand side. This region of high pressure grows as the returning wave propagates towards the vortex and attains values close to that behind the incident shock wave and reaches a value of 1.60 bar. There are no significant pressure changes on the left-hand side of the vortex due to the shock–vortex interaction. By 240 *μ*s (Figure 10(1)) the high-pressure region behind the returning diffracted shock wave is attenuating. There is very little change in the shape of the vortex, indicating a very weak shock–vortex interaction.

#### 5.2.3. CFD Results

**Figure 11.**
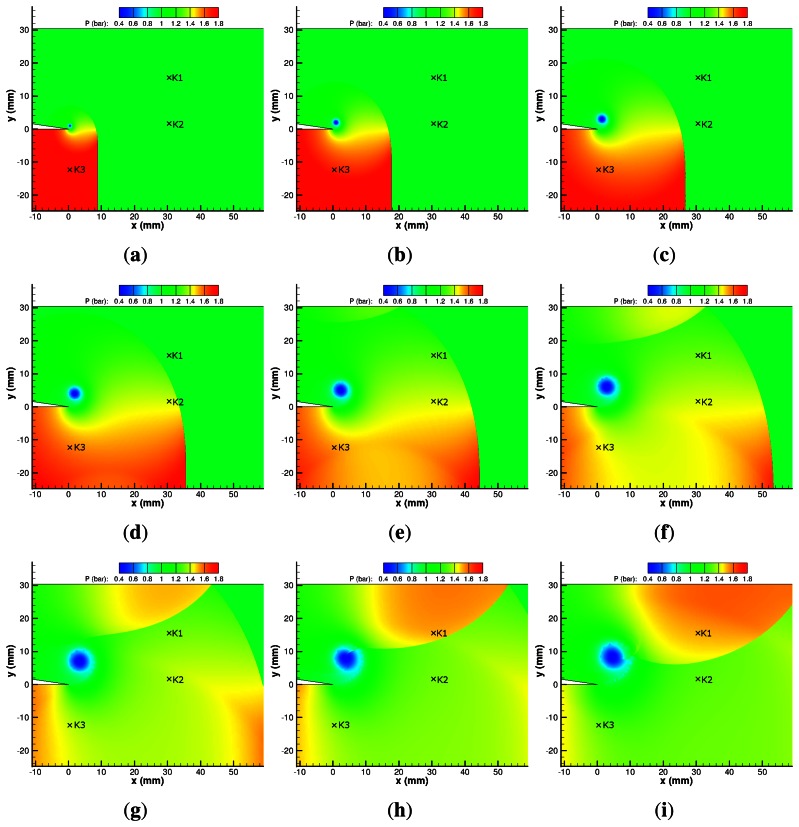

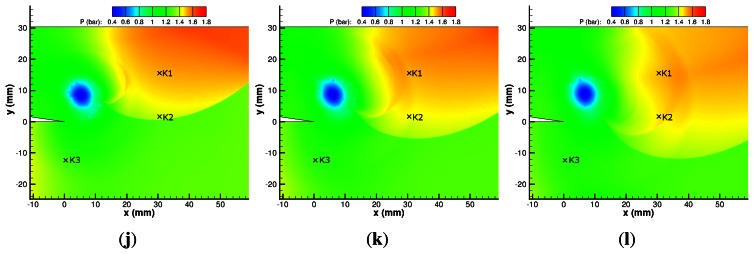
CFD pressure map of M*_i_* = 1.28 shock diffraction process. (**a**) 20 *μ*s; (**b**) 40 *μ*s; (**c**) 60 *μ*s; (**d**) 80 *μ*s; (**e**) 100 *μ*s; (**f**) 120 *μ*s; (**g**) 140 *μ*s; (**h**) 160 *μ*s; (**i**) 180 *μ*s; (**j**) 200 *μ*s; (**k**) 220 *μ*s; (**l**) 240 *μ*s.

The numerical pressure profile is presented in Figure 11. The numerical results qualitatively agree with the PSP measurements; however, there are some discrepancies. The simulated pressure behind the incident shock is slightly higher than the PSP measurements, albeit within experimental error. This is likely to be due to the response time of the PSP layer rather than an over-prediction by the simulation.

The reflected expansion wave can be seen to propagate upstream and is reflected from the bottom wall of the test section in Figure 11(d). The vortex is a clear region of low pressure, with pressures as low as 0.23 bar. This value is significantly lower than that measured in the experimental results.

The diffracted shock wave is clearly losing strength along its length from the triple point (the intersection of the contact surface, the incident shock and the diffracted shock). The diffracted portion of the shock causes hardly any pressure change above the splitter. The diffracted shock wave is reflected from the top wall of the test section and it is clear that it differs in strength along its length. The main vortex grows in size until 120 *μ*s (Figure 11(f)) and remains at approximately a constant size from then on, including after the shock–vortex interaction.

The shock–vortex interaction, shown in Figure 11(i) onwards, appears to be very weak and has little impact on the pressure profile. As is to be expected, the pressure varies slowly across the shear layer (owing to its steady curvature) and as such there is very little information available about it.

The reflected diffracted shock wave, returning from the top wall of the test section in Figure 11(g) to 11(l), gives a pressure of up to 1.60 bar, agreeing almost perfectly with the experimental values.

### 5.3. M_i_ = 1.55 Results

#### 5.3.1. Transducer Results

**Figure 12.**
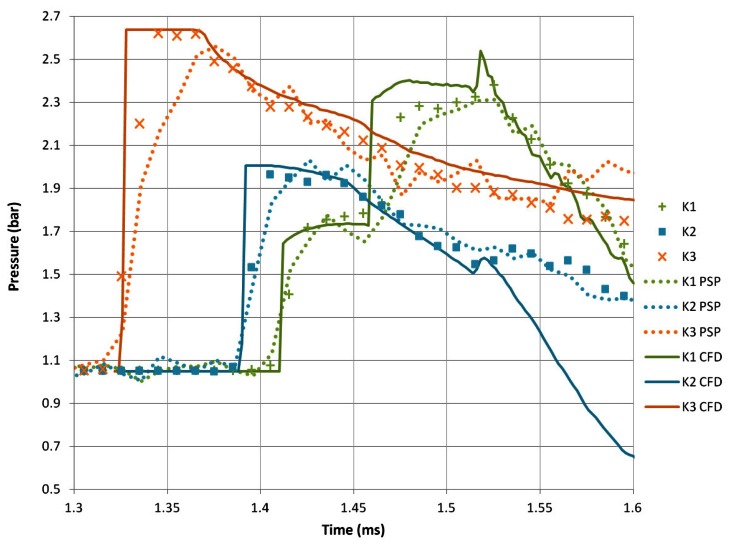
Pressure transducer measurements of M*_i_* = 1.55 shock diffraction process around sharp geometry.

The final pressure history for sharp geometries is shown in Figure 12. **K3** shows that the initial pressure rise is the highest recorded, at 2.62 bar with a similar attenuation to those seen in the previous cases. **K1** shows a similar two-stage pressure rise as the previous case, leading to a maximum of 2.38 bar. **K2** shows no secondary pressure rise, unlike the previous case. The maximum pressure reading from **K2** is 1.96 bar. This shows a fundamental change in the pressure history. This is likely to be due to the higher induced velocity in the expansion region and behind the incident shock wave in general. This increase in velocity has slowed the progress of the returning diffracted shock wave before the vortex is conducted away, therefore eliminating the second pressure rise within the time frame of Figure 12. The PSP measurements compare well with both numerical and virtual transducers, albeit with a slower response time. The highest pressure measured by the PSP is slightly lower than the transducers, whereas the decreases in pressure are very well represented in terms of both magnitude and time.

There are some large discrepancies between experimental and virtual transducers at this Mach number. After the initial shock wave, the numerical values of **K3** are slightly higher than the measured values from the Kulite transducers. The second pressure jump seen in the **K1** measurements arrives slightly earlier in the numerical simulation than in the real measurements. This is not a significant error as the actual transducers are finite in size, whereas the virtual transducers are point measurements at the centre of the real transducer location. The most significant discrepancy is seen between the real and numerical **K2** transducer. The corresponding PSP results do not show the same trend as the numerical simulations, leading to the conclusion that the numerical results have over-predicted the influence of the vortex after the shock–vortex interaction, causing a large decrease in pressure at the **K2** location.

#### 5.3.2. PSP Results

**Figure 13.**
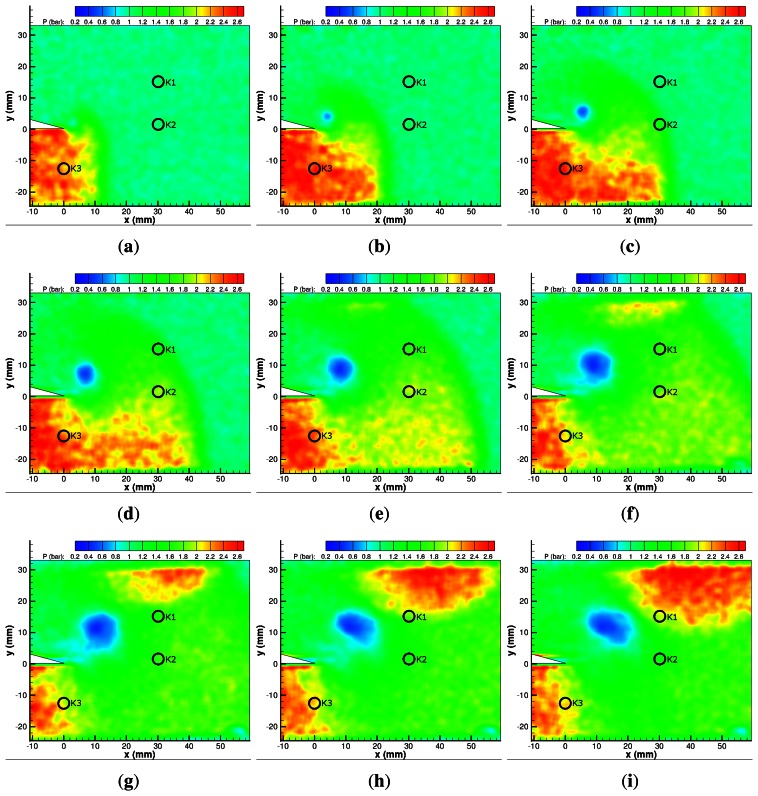

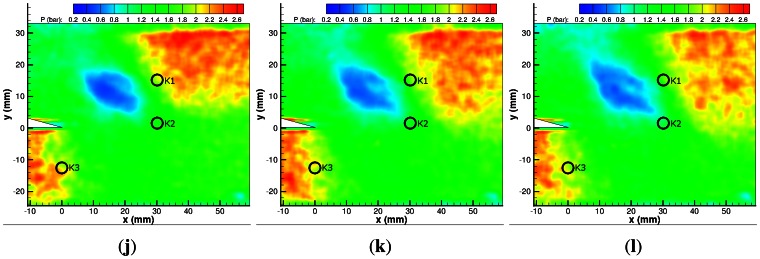
PSP map of M*_i_* = 1.55 shock diffraction process. (**a**) 20 *μ*s; (**b**) 40 *μ*s; (**c**) 60 *μ*s; (**d**) 80 *μ*s; (**e**) 100 *μ*s; (**f**) 120 *μ*s; (**g**) 140 *μ*s; (**h**) 160 *μ*s; (**i**) 180 *μ*s; (**j**) 200 *μ*s; (**k**) 220 *μ*s; (**l**) 240 *μ*s.

The final PSP results of the sharp geometry are presented in Figure 13. The pressure rise behind the shock is measured as 2.54 bar. This value compares reasonably well with the inviscid theory based on M*_ie_*, which gives a pressure rise of 2.64 bar. The vortex core is easily identifiable, as is the reflected expansion wave and its associated pressure drop. The lowest pressure measured in the vortex core is 0.40 bar. The pressure gradually increases from the vortex core, with no discontinuities nearby.

**Figure 14.**
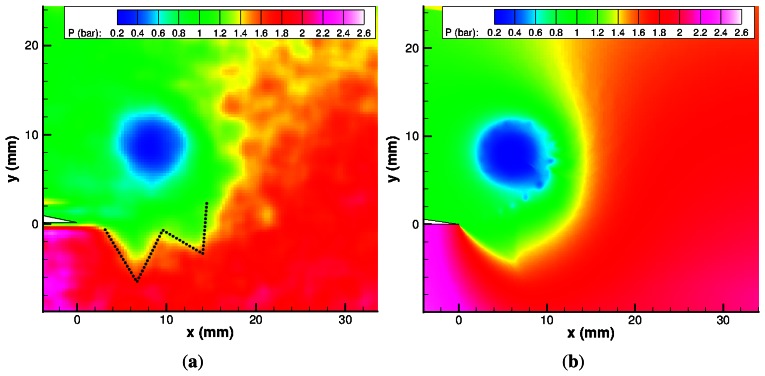
Experimental and numerical pressure map showing the lambda structures underneath the shear layer at 100 *μ*s. (**a**) PSP close-up; (**b**) CFD close-up.

Figures 13(e) and 13(f) begin to show the initial two lambda shock structures underneath the shear layer. This can be seen more clearly by changing the colour map and zooming in (see Figure 14(a)). The returning shock wave propagates as before, with an increase in pressure on the right-hand side of the vortex. The slowed returning shock wave can be seen on the PSP figures from Figure 13(j) onwards as an increase in pressure close to the vortex core. There is a pressure increase on the other side of the vortex core (approximately 210° around the vortex), which is not as well defined as a discontinuity, possibly due to its lower strength.

The main vortex is heavily distorted during the shock–vortex interaction. The once circular vortex has now been stretched to form an elliptical shape aligned at approximately 45° to the horizontal.

#### 5.3.3. CFD Results

**Figure 15.**
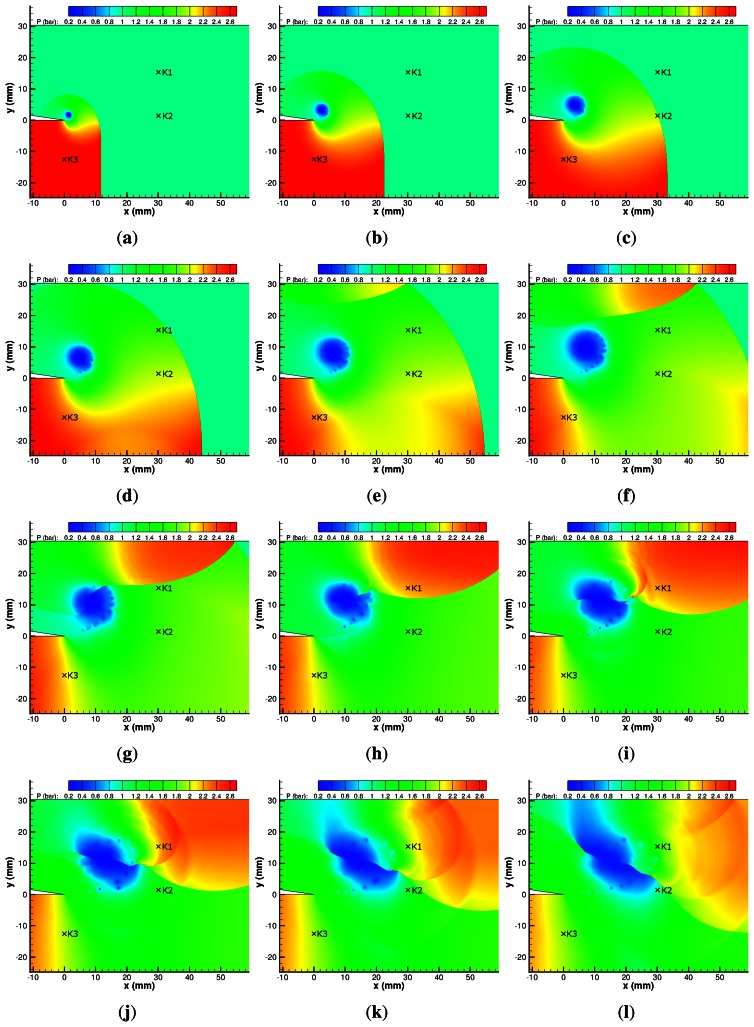
CFD pressure map of M*_i_* = 1.55 shock diffraction process. (**a**) 20 *μ*s; (**b**) 40 *μ*s; (**c**) 60 *μ*s; (**d**) 80 *μ*s; (**e**) 100 *μ*s; (**f**) 120 *μ*s; (**g**) 140 *μ*s; (**h**) 160 *μ*s; (**i**) 180 *μ*s; (**j**) 200 *μ*s; (**k**) 220 *μ*s; (**l**) 240 *μ*s.

The numerical simulations of a M*_i_* = 1.55 shock diffracting around a sharp corner are presented in Figure 15. The same flow features seen in the previous case are well represented, with the noticeable inclusion of the embedded shock wave between the shear layer and the vortex core, which can be faintly distinguished from Figure 15(c) onwards. The predicted pressure in the vortex core is significantly lower than the experimental results at 0.12 bar.

The predicted maximum pressure due to the returning shock wave is 2.41 bar, which agrees well with the experimental results (Figures 13(h) and 15(h)). The diverging acoustic waves generated by the interaction of the returning shock with the shear layer instabilities (Figure 15(j)) appear significantly stronger in the M*_i_* = 1.55 case.

The shock structure created by the shock-vortex interaction is very sharply defined in Figures 15(k) and 15(l). The position of the interaction agrees well with the PSP results at the same time step; however, the PSP results are obviously not as sharply defined.

In the latter images for Figure 15, the low-pressure region associated with the vortex appears to be spreading quickly along the upstream side of the shock wave system created by the shock–vortex interaction. The scale of this expansion region is much larger than that seen in the PSP results (Figures 13(l) and 15(l)). This larger expansion region explains the large discrepancy between the real and numerical transducers seen in Figure 12.

The numerical results fail to adequately resolve the lambda shock structure seen in the expansion region (see Figure 14(b)). This is due to the adaptive grid technique as it poorly resolves this structure. Results with uniform grids capture this phenomena well [28], although the computational cost can be very high.

### 5.4. Uncertainty and Repeatability

The uncertainty calculation for the PSP results is rather involved. To calculate the sensitivity coefficient, we first rearrange (1) for *P*/*P_ref_* and differentiate, giving:

(2) $$\frac{\partial \frac{P}{P_{\text{ref}}}}{\partial \frac{I_{\text{ref}}}{I}}=\frac{1}{\gamma B}{\left(\frac{\frac{I_{\text{ref}}}{I}-A}{B}\right)}^{\frac{1}{\gamma }-1}$$

Equation (2) can then be multiplied by the shot noise (shot noise is random statistical noise present when measuring any signal electrically) estimation (*σ_shot_*) and the calibration error (*σ_cal_*) to give us a bias in *P*/*P_ref_*. The random error is estimated from the uniform region behind the incident shock wave for all three repeats of each test condition. There is a random component of the bias error that accounts for the shot noise and excitation light intensity changes. As three sequences of images are ensemble averaged to give the final results, this shot noise error is divided by $\sqrt{3}$. Multiplying by *P_ref_* gives us the bias in *P*. The total uncertainty of the Kulite transducers used for calibration must also be considered in this analysis. The Kulite uncertainty consists of a component due to the ringing-type response to a step function input, a noise component and a random error in maximum pressure measured. The total uncertainty, Ψ, can then be estimated using:

(3) $$\Psi =\sqrt{\Psi_{\text{kulites}}^{2}+\sigma_{\text{random}}^{2}+\sigma_{\text{bias}}^{2}}$$

where

(4) $$\sigma_{\text{bias}}=P_{\text{ref}}\sqrt{{\left(\frac{\partial \frac{P}{P_{\text{ref}}}}{\partial \frac{I_{\text{ref}}}{I}}\times \sigma_{\text{shot}}\right)}^{2}+{\left(\frac{\partial \frac{P}{P_{\text{ref}}}}{\partial \frac{I_{\text{ref}}}{I}}\times \sigma_{\text{cal}}\right)}^{2}}$$

Table 2 shows the uncertainty estimation for the PSP results. The total uncertainty appears to be relatively constant for both Mach numbers, implying that the bias error is dominant. Table 2 shows that the shot noise is the dominant source of error in this experiment. This is not surprising, given the very short acquisition time.

## 6. Conclusions

Thin layer chromatography-based pressure-sensitive paint has been for the first time successfully applied to give quantitative measurements of the static pressure during the shock diffraction process. These results are one of the few successful attempts to measure a truly transient process using a camera rather than a PMT. The rise in pressure due to the shock wave and the decrease in pressure are well captured. There is some disagreement between the pressure measured in the vortex core and that of the numerical results. The spatial resolution of the numerical results is significantly higher than the experimental results, meaning that the exact centre of the vortex (*i.e.*, the cell of lowest pressure) can be captured more accurately.

The reflected expansion wave can be seen propagating away from the splitter tip at all three test conditions. In the M*_i_* = 1.55 case there are faint traces of lambda wave structures underneath the shear layer. These lambda structures are not very strong as they are poorly resolved in the pressure measurements. The pressure measured in the expansion region is approximately 1 bar regardless of the incident shock Mach number, validating one of the assumptions of Sun and Takayama's theory [17].

The reflection of the diffracting shock wave from the top wall of the test section can be seen in both cases as a secondary increase in pressure. The pressure reached in this region has almost the same magnitude as the pressure behind the incident shock wave for both cases.

The shock–vortex interaction, specifically the progress of the shock on the right-hand side of the vortex, is captured well and represents the sharpest discontinuity measured by the PSP. This is not surprising, as this shock wave moves significantly more slowly than the incident shock and produces a similar magnitude of pressure jump. The shock on the left-hand side of the vortex is not well captured by the PSP, indicating that it is significantly weaker. The distortion to the main vortex, caused by the returning diffracted shock wave, is reflected in the PSP results. For the M*_i_* = 1.28 case, the distortion is barely noticeable and the vortex remains a circular region of low pressure. However, the subsequent case shows that the vortex is heavily distorted with a discontinuity near the vortex core. The pressure spike seen by Skews *et al.* [29] is not seen in either the numerical or experimental results.

The *low* spatial resolution of the PSP results and the relatively high degree of spatial noise mean that no information can be gathered about the shear layer. It would be expected that, initially, where the shear layer is straight, the pressure on either side would be equal, meaning that PSP would not give significant information.

There is a significant degree of spatial noise present in the experimental results. This is primarily due to the shot noise. The spatial noise can only be reduced by ensemble averaging multiple runs as it consists of random fluctuations. Future improvements in CCD technology should also reduce this problem. Despite the high level of spatial noise, the experimental and numerical results agree within experimental error, with the exception of the exact vortex core pressure. The PSP measurements also show that the influence of the low-pressure region after the M*_i_* = 1.55 shock–vortex interaction is not as strong as the numerical predictions. This is likely a numerical error rather than the PSP not responding quickly enough, as the results of Gregory and Sullivan show that PSP responds to a decrease in pressure more quickly than to an increase in pressure [30]. Gregory and Sullivan's conclusion is demonstrated in these results, as the increase in pressure behind the incident shock wave is not as well captured as the decrease in pressure in the following frames.

## Figures and Tables

**Table 1.** PSP calibration constants.

*M_ie_*	***A***	***B***	*γ*	R^2^
1.28	−284.001	285.002	2.736 × 10^−4^	0.934
1.55	−511.035	512.037	1.590 ×10^−4^	0.930

**Table 2.** PSP uncertainty.

**M***_ie_*	***σ****_shot_* ***I****_ref_*/***I***	***σ****_cal_* ***I****_ref_*/***I***	***σ****_random_* ***I****_ref_*/***I***	**Ψ***_Kulites_*	**Total Pressure** **Uncertainty Ψ** (bar)
1.28	0.0291	0.00156	0.0045	0.0841	0.261
1.55	0.0287	0.00684	0.0022	0.1321	0.259
